# Supplementary material for: Box breathing or six breaths per minute: Which strategy improves athletes post-HIIT cardiovascular recovery?
Source: PLoS One. 2025 Nov 17;20(11):e0336615. doi: 10.1371/journal.pone.0336615 (PMC12622787; doi:10.1371/journal.pone.0336615)
Supplement: Attachment — Submitted filename: Reviewer_PONE-D-25-41258.docx [file pone.0336615.s003.docx]

The study sought to investigate a relevant topic by comparing the impact of three different breathing protocols on cardiovascular and perceptual recovery. The proposal is interesting, as it addresses an area of growing scientific and practical interest, especially in the context of physical performance and health. However, some adjustments are necessary to improve the clarity and consistency of the text.

**General Recommendations**

References should be formatted according to the Vancouver style, as required by the journal.

Objectives and hypotheses should be presented within the running text, rather than in separate subsections.

**Introduction**

Line 43: The sentence “...also how and how quickly the body responds to this load..” contains a repeated “how” and should be corrected.

Line 52: The term “psychological variables” requires clarification. Please specify which variables are considered (e.g., stress) and provide a brief description.

**Materials and Methods**

Exclusion criteria were not reported. If applied, please describe them.

Was there any control regarding the type of sport practiced by participants? Certain sports involving respiratory techniques could bias the sample.

Please provide more details about the randomization process, including the method used.

Clarify how respiratory control was adequately ensured during the procedures.

Were heart rate variability (HRV) data collected? If so, their inclusion could substantially enrich the discussion.

**Results**

Measures of dispersion for the mean difference are missing in Table 4.

**Discussion**

The paragraph addressing heart rate variability states:

“In addition, the difference detected in heart rate variability was found to be significant not only statistically but also in terms of effect size (η² = 0.127). In particular, Cohen's d = 0.907 obtained in the comparison between Box Breathing and 6 Breaths/min indicates a large difference. This supports the idea that the aforementioned breathing technique may have a significant effect on physiological stress.”

This section is confusing.

It is necessary to clarify whether HRV data were actually collected or whether only heart rate (HR) was measured. HRV indices, such as time-domain and frequency-domain parameters, could strengthen the interpretation of the findings.

If only HR was measured, please revise the terminology to avoid misleading readers by conflating HR with HRV.
