# Supplementary material for: Box breathing or six breaths per minute: Which strategy improves athletes post-HIIT cardiovascular recovery?
Source: PLoS One. 2025 Nov 17;20(11):e0336615. doi: 10.1371/journal.pone.0336615 (PMC12622787; doi:10.1371/journal.pone.0336615)
Supplement: Attachment — Submitted filename: Response to Reviewers.docx [file pone.0336615.s005.docx]

**Response to Reviewers**

We would like to thank the Academic Editor and both Reviewers for their valuable comments and suggestions. These comments significantly improved the quality and clarity of our article.

Below, we provide point-by-point responses to each comment.

Reviewer 1's comments are highlighted in yellow, and Reviewer 2's comments are highlighted in turquoise, followed by our responses and a summary of the changes made (**Previous version**, **Edited version).**

We hope that the revised manuscript meets the expectations of the reviewers and the journal. We sincerely appreciate the opportunity to revise and resubmit our work.

Kind regards,

Dr. Murat Kasap

Corresponding Author

**Reviewer #1:**

1) **References should be formatted according to the Vancouver style, as required by the journal.**

**Previous version**

**1a)** “The post-exercise recovery process plays a critical role in increasing training efficiency, maintaining high-level performance, and reducing the risk of injury in athletes (Zaccaro et al., 2018; Kumar et al., 2024)…….”

*References List*

**1b)**

Zaccaro, A., Piarulli, A., Laurino, M., Garbella, E., Menicucci, D., Neri, B., & Gemignani, A. (2018). How breath-control can change your life: A systematic review on psycho-physiological correlates of slow breathing. Front. Hum. Neurosci., 12, 353. <https://doi.org/10.3389/fnhum.2018.00353>

**1c)**

Kumar, S., Das, R., & Sharma, V. (2024). Effects of 6 breaths-per-minute breathing and box breathing techniques on anxiety, perceived stress, and physiological indicators: A randomized controlled trial. Complement. Ther. Clin. Pract., 54, 102150. <https://doi.org/10.1016/j.ctcp.2024.102150>

**Edited version**

**1a)** The post-exercise recovery process plays a critical role in increasing training efficiency, maintaining high-level performance, and reducing the risk of injury in athletes [1, 2].

*References List*

**1b)**

1. Zaccaro A, Piarulli A, Laurino M, Garbella E, Menicucci D, Neri B, et al. How breath-control can change your life: A systematic review on psycho-physiological correlates of slow breathing. Front Hum Neurosci. 2018;12:353. <https://doi.org/10.3389/fnhum.2018.00353>

**1c)**

2. Kumar S, Das R, Sharma V. Effects of 6 breaths-per-minute breathing and box breathing techniques on anxiety, perceived stress, and physiological indicators: A randomized controlled trial. Complement Ther Clin Pract. 2024;54:102150. <https://doi.org/10.1016/j.ctcp.2024.102150>

**Note: The "Vancouver style" correction given above has been applied to the entire article.**

2) **Objectives and hypotheses should be presented within the running text, rather than in separate subsections.**

**Previous version**

**2a)**…….. perceived exertion following high-intensity interval training (HIIT) are quite limited.

**Study objective and hypotheses**

Building on this gap, this study aimed to investigate the acute effects of three different breathing protocols (Box Breathing, 6 Breaths per Minute, Spontaneous Breathing) on physiological recovery during high-intensity interval (HIIT) cycling exercise on a spin bike in sports science students of similar age and physical capacity.

**Hypotheses:**

H1: Box Breathing and 6 Breaths per Minute protocols will positively affect heart rate recovery and perceived exertion (Borg RPE scores) compared to spontaneous breathing.

H2: Controlled breathing techniques will reduce perceived exertion (Borg RPE scores) more effectively than spontaneous breathing.

H3: 6 Breaths per Minute may have a stronger effect on cardiovascular and perceptual recovery metrics than the Box Breathing protocol.

**Edited version**

The hypotheses tested in the study were as follows: *H1:* Box Breathing and 6 Breaths per Minute protocols will positively affect heart rate recovery and perceived exertion (Borg RPE scores) compared to spontaneous breathing. *H2:* Controlled breathing techniques will reduce perceived exertion (Borg RPE scores) more effectively than spontaneous breathing. *H3:* 6 Breaths per Minute may have a stronger effect on cardiovascular and perceptual recovery metrics than the Box Breathing protocol.

**Note: Objectives and hypotheses are presented in both the summary and introduction sections as a continuation of the text.**

3) **Introduction**

**3a) Line 43: The sentence “...also how and how quickly the body responds to this load..” contains a repeated “how” and should be corrected.**

**Previous version**

**3a)** Modern training science considers recovery to be one of the fundamental determinants of performance, taking into account not only the training load but also how and how quickly the body responds to this load.

**Edited version**

**3a)** Modern training science considers recovery to be one of the fundamental determinants of performance, taking into account not only the training load but also the manner and speed with which the body responds to it."

**3b) Line 52: The term “psychological variables” requires clarification. Please specify which variables are considered (e.g., stress) and provide a brief description.**

**Previous version**

Studies on the physiological effects of breathing exercises have reported positive results in cardiopulmonary parameters such as increased vagal tone, improved heart rate variability (HRV), and improved baroreflex sensitivity (Lehrer & Gevirtz, 2014; Laborde, 2019). Slow and controlled breathing suppresses sympathetic activity and increases parasympathetic responses, leading to significant improvements in heart rate, blood pressure, and psychological stress levels (Morgan et al., 2024; Hopper et al., 2019).

**Edited version**

"Studies on the physiological effects of breathing exercises have reported positive results in cardiopulmonary parameters such as increased vagal tone, improved heart rate variability (HRV), and improved baroreflex sensitivity [7,8]. Slow and controlled breathing suppresses sympathetic activity and increases parasympathetic responses, leading to significant improvements in heart rate, and blood pressure. This physiological shift is directly associated with a reduction in psychological stress, which in this context refers to measurable decreases in perceived stress and state anxiety, as well as an improvement in overall emotional well-being [9,10,14]."

4) **Materials and Methods**
a) **Exclusion criteria were not reported. If applied, please describe them.**
b) **Exclusion criteria were not reported. If applied, please describe them.**
c) **Was there any control regarding the type of sport practiced by participants? Certain sports involving respiratory techniques could bias the sample.**
d) **Please provide more details about the randomization process, including the method used.**
e) **Clarify how respiratory control was adequately ensured during the procedures.**
f) **Were heart rate variability (HRV) data collected? If so, their inclusion could substantially enrich the discussion.**

**Previous version**

**Participants**

Forty students (25 males, 15 females; age: 20.95 ± 1.75 years; BMI: 22.97 ± 3.25 kg/m²) from the Faculty of Sports Sciences at Bartın University voluntarily participated in the study. Participants were individuals who engaged in at least 2 days per week of physical activity at a level that included aerobic or anaerobic exercise. All participants were familiar with bicycle ergometer training and had no known cardiopulmonary or orthopaedic conditions. The study was approved by the Bartın University Ethics Committee (Approval No: 2025-SBB-0244) and conducted in accordance with the Helsinki Declaration. All participants were of legal age and signed written informed consent before the study.

**Application procedure**

A randomized crossover design was employed in the study. Each participant……

**Edited version**

**Participants**

**4 a-b-c)** Forty students from Bartın University's Faculty of Sports Sciences (25 male, 15 female; age: 20.95 ± 1.75 years; BMI: 22.97 ± 3.25 kg/m²) voluntarily participated in the study. The participants were athletes who engaged in physical activity comprising at least two days of aerobic or anaerobic exercise per week, had no prior experience with breathing exercises or meditation-based activities, and were not currently practicing them. All participants were familiar with cycle ergometer training and had no known cardiopulmonary or orthopedic disorders. The participants were not practicing any techniques such as single-breath diving, breathing exercises, or meditation. The study was approved by the Bartın University Ethics Committee (Approval No: 2025-SBB-0244) and conducted in accordance with the Declaration of Helsinki. All participants were of legal age and provided written informed consent prior to the study.

**Inclusion Criteria:**

-Voluntary participation.

-Age 18-24.

-Student in the Faculty of Sports Sciences.

-Must not have previously engaged in, and must not be currently engaging in, any breathing exercises or meditation-based activities.

**Exclusion Criteria:**

-Failure to meet one or more of the inclusion criteria listed above was defined as an exclusion criterion.

**4 d) Randomization**

The order of the three breathing protocols was randomized for each participant using a computer-generated random sequence ([www.randomizer.org)](http://www.randomizer.org))."

The order was as follows:

Session 1

37, 36, 11, 27, 15, 38, 1, 29, 24, 7, 4, 2, 34, 23, 33, 6, 14, 5, 40, 21, 12, 32, 13, 3, 16, 25, 31, 35, 18, 26, 30, 17, 20, 9, 8, 28, 19, 39, 10, 22

Session 2

32, 2, 19, 37, 33, 11, 15, 21, 29, 39, 28, 6, 40, 31, 18, 27, 22, 16, 3, 38, 12, 13, 26, 4, 30, 35, 36, 17, 34, 5, 20, 10, 24, 7, 25, 14, 23, 8, 9, 1

Session 3

5, 12, 15, 10, 28, 13, 4, 36, 25, 31, 18, 19, 2, 7, 35, 39, 32, 3, 40, 14, 26, 23, 22, 33, 9, 38, 16, 29, 8, 17, 6, 34, 11, 20, 1, 37, 24, 30, 21, 27

**4 e) Clarify how respiratory control was adequately ensured during the procedures.**

Specially designed video slides for the three distinct breathing protocols were projected onto the wall in front of the participants in the spin bike room. The test flow guided the athletes both visually and auditorily.

**Figure 1.** Example (4-4-4-4) square breathing video presentation stage visuals

Respiratory control was rigorously ensured through a standardized, audiovisual guidance system that minimized participant discretion and ensured strict adherence to the prescribed breathing protocols. The procedure was as follows:

Specially designed video slides for each of the three distinct breathing protocols were projected onto the wall in front of the participants. The entire test flow was governed by this system, which provided simultaneous visual and auditory commands.

Prior to each testing session, participants were familiarized with the specific breathing protocol they would perform to ensure comprehension.

The control process for a single cycle (e.g., the 4-4-4-4 square breathing protocol) was as follows:

1- The session began with a 30-second exercise bout initiated by an audible "start test" command. A countdown timer was displayed. At the end of the exercise, an auditory cue announced the transition to a 60-second rest/breathing period. During this period, each phase of the breathing cycle was precisely guided:
   2-3-4-5 An audible "inhale" cue was given, accompanied by a visual timer counting down 4 seconds. This was sequentially followed by "hold breath" (4 seconds), "exhale" (4 seconds), and again "hold breath" (4 seconds). This cycle was repeated throughout the 60-second rest period. A verbal and visual warning was given at the 55-second mark to prepare for the next exercise bout.

Moreover "Researchers visually monitored each participant's chest movement and provided immediate verbal feedback to ensure adherence to the target breathing rhythm throughout the recovery period."

The sequence of 30-second exercise followed by the 60-second guided breathing period was repeated until the total session duration of 15 minutes was completed.

6-The test was terminated automatically upon reaching the 15-minute mark with an audible "test finished" command, immediately followed by the display of the Borg Scale for participant rating.

This method of automated, step-by-step audiovisual guidance ensured that all participants followed the exact same rhythm and timing for each breath, thereby guaranteeing adequate and consistent respiratory control throughout the procedures.

**4f)** **Were heart rate variability (HRV) data collected? If so, their inclusion could substantially enrich the discussion.**

**Previous version**

This mechanism differs from findings in low-intensity studies and suggests that the effects of breathing techniques may vary depending on the exercise modality. Future studies should validate this hypothesis using end-tidal CO₂ and HRV measurements.

In addition, the difference detected in heart rate variability was found to be significant not only statistically but also in terms of effect size (η² = 0.127). In particular, Cohen's d = 0.907 obtained in the comparison between Box Breathing and 6 Breaths/min indicates a large difference. This supports the idea that the aforementioned breathing technique may have a significant effect on physiological stress.

**Edited version**

**"Were heart rate variability (HRV) data collected?" The paragraph was updated to avoid ambiguity, and the Parameters Measured and Observed in the Study section was added to the Materials and methods section.**

The observed differences were substantiated by large effect sizes for both final heart rate (η² = 0.127; Box vs. 6 bpm Cohen's d = 0.907) and perceived exertion (η² = 0.374; Box vs. Spont. d = 1.525, Box vs. 6 bpm d = 1.694), underscoring the substantial physiological and perceptual impact of the box breathing technique. In contrast, no significant difference was found in recovery time to baseline heart rate between protocols, which contrasts with studies reporting facilitatory effects of similar techniques on recovery [21,23]. This discrepancy may be attributed to differences in exercise intensity, participant characteristics, or protocol duration, highlighting the need for future studies with standardized long-term applications on homogeneous groups to clarify these effects. The moderate effect size for recovery time between Box Breathing and 6 bpm (Cohen's d = 0.502), though statistically non-significant, warrants further investigation into the potential benefits of rhythmic breathing like 6 bpm.

**Measured and Observed Parameters**

The following primary outcome variables were assessed for each breathing protocol:

•	Initial Heart Rate (HR): HR measured at the end of the 10-minute warm-up period.

•	Post-Exercise Heart Rate (HR): HR measured immediately upon completion of the final HIIT interval.

•	Heart Rate Recovery Time: The time elapsed from the cessation of exercise until HR returned to the individual's pre-exercise baseline (Initial HR) value.

•	Rating of Perceived Exertion (RPE): Assessed using the Borg RPE scale (6-20) immediately after each test session.

**5 Result**

**Measures of dispersion for the mean difference are missing in Table 4.**

**Previous version**

| **Table 4.** Post-Hoc Test (Tukey HSD) Results | | | |
| --- | --- | --- | --- |
| **Variable** | **Comparison** | **Mean Difference** | **p-value** |
| Final Heart Rate (beats/min) | 6 Breaths vs Box Breathing | +9.87 | p < 0.001*** |
|  | 6 Breaths vs Control | +4.28 | 0.179 |
|  | Box Breathing vs Control | -5.60 | 0.054 |
| Perceived Exertion (Borg Scale) | 6 Breaths vs Box Breathing | -2.03 | p < 0.001*** |
|  | 6 Breaths vs Control | 0.00 | 1.000 |
|  | Box Breathing vs Control | +2.03 | p < 0.001*** |

**Edited version**

| **Table 4.** Post-Hoc Test (Tukey HSD) Results | | | | |
| --- | --- | --- | --- | --- |
| **Variable** | **Comparison** | **Mean Difference** | **95%CI** | **p-value** |
| Final HR (bpm) | Box Breathing vs 6 bpm | +9.87 | [6.32, 13.42] | **< 0.001** |
|  | 6 bpm vs Spont. | +4.28 | [-1.97,10.53] | 0.179 |
|  | Box Breathing vs Spont. | -5.60 | [-11.32, 0.12] | 0.054 |
| Perceived Exertion (Borg Scale) | Box Breathing vs 6 bpm | -2.03 | [-2.51, -1.55] | **< 0.001** |
|  | 6 bpm vs Spontaneous | 0.00 | [-0.57, +0.57] | 1.000 |
|  | Box Breathing vs Spont. | +2.03 | [+1.48, +2.58] | **< 0.001** |

Post-hoc pairwise comparisons were performed using Tukey's HSD test to identify specific differences between respiratory protocols. Statistically significant p-values (p < 0.001) are presented in bold. Spont.: Spontaneous.

**6 Discussion**

**The paragraph addressing heart rate variability states:**

**“In addition, the difference detected in heart rate variability was found to be significant not only statistically but also in terms of effect size (η² = 0.127). In particular, Cohen's d = 0.907 obtained in the comparison between Box Breathing and 6 Breaths/min indicates a large difference. This supports the idea that the aforementioned breathing technique may have a significant effect on physiological stress.”**

**This section is confusing.**

**It is necessary to clarify whether HRV data were actually collected or whether only heart rate (HR) was measured. HRV indices, such as time-domain and frequency-domain parameters, could strengthen the interpretation of the findings.**

**If only HR was measured, please revise the terminology to avoid misleading readers by conflating HR with HRV.**

**Edited version**

The solution to this ambiguity is at the top of the page. See (4f Edited)

**Reviewer #2:**

**1. Determination of HRmax**

**The manuscript states that participants performed HIIT based on 85–95% of their maximum heart rate (HRmax), but does not specify how this HRmax was determined. It is essential to clarify whether a direct exercise stress test or a predictive formula (e.g., 220 - age) was used, as this directly impacts the accuracy of the exercise prescription.**

**Previous version**

There was no information including the HRmax calculation method

**Edited version**

The necessary explanation was added to the method section.

**Determination of HRmax**

Maximum heart rate (HRmax) was estimated using the Tanaka formula: HRmax = 208 − (0.7 × age), which has been shown to be more accurate than the traditional 220 − age formula, particularly in healthy adults [16]. This estimation guided the target training zone (85–95% HRmax) during HIIT intervals.

**2. Standardization of Terminology**

**There are inconsistencies in how the six-cycles-per-minute breathing protocol is referred to: "six breaths per minute," "6 breaths/min," "6 breaths/minute." It is suggested to standardize this to "6 bpm" (breaths per minute) throughout the manuscript, maintaining consistency and clarity.**

**Previous version**

The term "6 breath cycles per minute" was used interchangeably throughout the article as "six breaths per minute," "6 breaths/min," "6 breaths/minute."

**Edited version**

It was standardized as " 6 bpm" throughout the article.

**3 Discussion – Practical Applicability**

**The discussion could be improved with greater emphasis on the practical implications of the findings, especially for coaches, physical trainers, and physiologists. For example, how can the 6 bpm breathing protocol be easily implemented in sports settings? What is the best way to instruct athletes? Should it be applied immediately after exercise or in separate sessions?**

**Previous version**

Practical Implications was not included in the Discussion section.

**Edited version**

The "Practical Applicability" description identified and suggested has been added as a subheading to the Discussion section.

**Practical Implications**

The findings of this study offer valuable practical applications for coaches, athletes, and fitness professionals. According to our results, the 6 breaths/min breathing protocol (5-second inhale, 5-second exhale) is a more effective and less stressful strategy for post-HIIT recovery compared to Box Breathing (4-4-4-4). This technique can be easily implemented in training settings: coaches can instruct athletes to begin the 6 breaths/min rhythm during rest intervals between sets or immediately upon finishing a session, using simple verbal cues ("Breathe in... two... three... four... five... Now breathe out... two... three... four... five...") or a visual metronome to maintain the pace. The protocol's simplicity allows for rapid adoption without the need for specialized equipment. In contrast, practitioners should be cautious when prescribing Box Breathing immediately after high-intensity exercise, as it may inadvertently increase physiological and perceptual stress, potentially delaying recovery. For optimal results, the regular application of the 6 breaths/min technique during inter-set rest periods or post-training is recommended.

**4. Limitations**

**The study acknowledges limitations such as its focus on acute effects and the sample size of young university students. However, it is recommended to emphasize that the generalizability of the results is limited and that the effects may vary depending on the intensity of the exercise, the sport, and the training level of the participants.**

**Previous version**

**Limitations and Future Studies**

One of the main limitations of this study is that it only evaluates the acute effects of breathing techniques. The effects of long-term applications (e.g., 4–8 weeks) on autonomic nervous system adaptations are unknown. Chronic studies should monitor changes in parameters such as HR recovery and HRV and include additional physiological markers such as baroreflex sensitivity (Dupuy et al., 2018). Additionally, the use of respiratory rate monitors to standardise participants' breathing patterns would enhance data reliability.

Future research should compare the effects of controlled breathing protocols across different exercise intensities and durations.

Different age groups and resting heart rate levels may provide different assessments and perspectives on the evaluation of heart rate recovery (HRR) and parasympathetic activity..

**Edited version**

The limitations section, which was suggested as a deficiency, has been expanded.

**Limitations and Future Studies**

This study has several limitations that should be considered. Firstly, the findings are constrained by their acute nature, capturing only the immediate effects of a single application of the breathing protocols. The long-term adaptations and efficacy of sustained practice (e.g., over 8-12 weeks) remain unknown and represent a crucial area for future investigation.

Secondly, the generalizability of the results is limited by the specific sample characteristics. The participants were young, healthy, and physically active sports science students. Consequently, these findings may not be directly applicable to older adults, elite athletes, sedentary individuals, or populations with different fitness levels or health conditions. The effectiveness of these breathing strategies is likely influenced by factors such as age, baseline fitness, and training status, which warrants further exploration.

To address these limitations, future chronic studies should monitor changes in parameters such as HR recovery and heart rate variability (HRV), incorporating additional physiological markers like baroreflex sensitivity [3]. Furthermore, research should compare the effects of these protocols across different exercise intensities, durations, and population groups. The use of respiratory rate monitors to standardize breathing patterns would also enhance the reliability of future data.

**5. Redundancy and Style**

**The discussion presents some repetitions and could be more concise in certain sections. A revision is suggested for greater objectivity, without compromising the depth of the analysis.**

**Edited version**

Updates and corrections have been made throughout the article, taking into account the relevant sections and the warnings and suggestions. (Please see the full article in its updated version.)

6 **Summary Recommendations**
a) **Include clear details on the method for determining HRmax.**

**Previous version**

There was no information in the previous version

**Edited version**

The necessary explanation was added to the method section.

**Determination of HRmax**

Maximum heart rate (HRmax) was estimated using the Tanaka formula: HRmax = 208 − (0.7 × age), which has been shown to be more accurate than the traditional 220 − age formula, particularly in healthy adults [16]. This estimation guided the target training zone (85–95% HRmax) during HIIT intervals.

b) **Standardize the nomenclature to "6 bpm" throughout the text.**

**Previous version**

The term "6 breath cycles per minute" was used interchangeably throughout the article as "six breaths per minute," "6 breaths/min," "6 breaths/minute."

**Edited version**

It was standardized as "6 bpm" throughout the article.

c) **Streamline the discussion, reducing repetitions.**

**Previous version**

1. **Discussion**

The aim of this study was to compare the effects of three different breathing protocols (spontaneous breathing, box breathing, and 6 breaths per minute) on heart rate, recovery time, and perceived exertion after high-intensity interval training (HIIT) performed on a spin bike. The findings revealed that the Box Breathing protocol resulted in significantly higher heart rates and Borg scores compared to the other protocols. These findings suggest that breathing techniques may not only affect recovery processes but also the perceived physiological load during exercise.

First, participants who followed the Box Breathing protocol exhibited significantly higher heart rates after exercise compared to other protocols. This suggests that the protocol may have a significant physiological effect on the autonomic nervous system during or after exercise. However, the literature reports that slow and rhythmic breathing techniques generally activate the parasympathetic system and lower heart rate (Zaccaro et al., 2018). In our study, however, the Box Breathing protocol, consisting of a 4-4-4-4 cycle, was found to increase heart rate after exercise rather than decrease it. This may be related to the increase in diaphragmatic and thoracic pressure caused by prolonged breath-holding phases (Lehrer & Gevirtz, 2014; Michael et al., 2016). Additionally, the increase in heart rate after exercise with Box Breathing may seem to contradict the literature suggesting that slow breathing techniques generally support parasympathetic activation (Zaccaro et al., 2018). However, this finding should be interpreted in the context of the high metabolic stress created by HIIT. Breath-holding phases (4 seconds) may have triggered a sympathetic response by causing arterial CO₂ accumulation (hypercapnia) during the increased metabolic demand post-exercise (Bernardi et al., 2001). Similarly, Michael et al. (2016) reported that breath-holding after submaximal exercise temporarily reduced HRV. This mechanism differs from findings in low-intensity studies and suggests that the effects of breathing techniques may vary depending on the exercise modality. Future studies should validate this hypothesis using end-tidal CO₂ and HRV measurements.

In addition, the difference detected in heart rate variability was found to be significant not only statistically but also in terms of effect size (η² = 0.127). In particular, Cohen's d = 0.907 obtained in the comparison between Box Breathing and 6 Breaths/min indicates a large difference. This supports the idea that the aforementioned breathing technique may have a significant effect on physiological stress.

On the other hand, it was observed that the Box Breathing protocol did not contribute significantly to recovery time. This finding differs from some studies that reported that it facilitates recovery (Buxton et al., 2024; Wang et al., 2023;	Yadav & Mutha, 2016). Possible reasons for this discrepancy include differences in exercise intensity, individual variables such as participants' age and experience level, and the application duration of the breathing protocol differing from that reported in the literature. Therefore, future studies should control for such variables and re-test the physiological effects of Box Breathing using long-term applications on more homogeneous samples.

Although the effect size (η² = 0.034) was small in terms of recovery time, the difference between Box Breathing and the 6 Breaths/min protocol was found to be moderate (Cohen’s d = 0.502). This result suggests that the potential effects of rhythmic breathing patterns, such as 6 Breaths/min, on the recovery process should be further investigated in future studies.

The 6 breaths/minute protocol, often referred to in the literature as resonance frequency breathing, has been shown to enhance cardiorespiratory synchronisation by increasing vagal activity (Kumar et al., 2024; Magnon et al., 2021). In our study, this protocol showed heart rate and Borg scores similar to those observed in spontaneous breathing. This finding is consistent with Morgan et al. (2024) who noted that slow breathing reduces anxiety but does not have a dramatic effect on post-exercise physiological recovery.

Although there was no statistically significant difference in terms of recovery time (p=0.128), a tendency toward delay in returning to baseline values was observed under box breathing conditions. With Cohen's d=0.502 indicating a moderate effect size, this finding may have practical significance (Lakens, 2013). For example, the ~8-second difference between 75.3 seconds (Box Breathing) and 67.5 seconds (6 breaths/min) for coaches could represent a critical time window in competitive sports. However, larger sample studies are needed to test the validity of this interpretation. This remains a topic of debate in the literature. While Joseph et al. (2005) suggested that slow breathing increases baroreflex sensitivity and contributes to circulatory stabilisation, Atlı et al. (2023) reported that such techniques may delay recovery in certain individuals. These findings highlight the possibility that the physiological effects of breathing techniques may vary depending on individual responses (Facioli et al., 2021).

The difference observed in the Borg Score in our study was supported not only statistically but also by a very high effect size (η² = 0.374). In particular, Box Breathing created much higher perceived exertion compared to both the Spontaneous and 6 Breaths/min protocols; these differences were calculated to be very large, with Cohen’s d values of 1.525 and 1.694. This indicates that breath-holding phases after high-intensity exercise create both physiological and psychological stress on participants.

A notable finding related to perceived exertion was that the Box Breathing protocol yielded significantly higher Borg scores than both the 6 breaths/minute and spontaneous breathing protocols. While this finding appears to contradict the results of Kumar et al. (2024), it should be interpreted with caution. Their study involved low intensity exercise, whereas our protocol followed moderate to high-intensity interval sessions. Additionally, the inclusion of breath-holding phases (box breathing) under high heart rate conditions may have increased psychological stress and perceived exertion in some participants ( Liang et al., 2023; Balban et al., 2023).

Finally, there are only a limited number of studies in the literature that directly evaluate the acute effects of breathing techniques on post-exercise physiological recovery (Bentley et al., 2023; Russo et al., 2017). Additionally, Garcia et al. (2024) highlighted that individuals with lower resting heart rate (RHR) exhibited faster HRR and stronger parasympathetic reactivation, while Danieli et al. (2014) emphasised that resting HRV values in middle-aged individuals were significantly positively correlated with early HRR post-exercise. These findings draw attention to the effects of resting heart rate and age on autonomic regulation and suggest the need for age-specific analyses.

In this context, our study contributes significantly to the field by comparing the effects of controlled breathing techniques on post-exercise heart rate and perceived exertion in young, physically active individuals.

2. **Conclusions and Recommendations**

The results of this study strengthen the assessment of different breathing protocols as indicators that may create significant differences in physiological recovery and perceived exertion after high-intensity interval training (HIIT).

**Compared to Spontaneous Breathing:**

- The Box Breathing protocol resulted in higher heart rate and perceived exertion.
- The 6 Breaths/Minute protocol led to lower physiological and perceptual loads.
- Although there was no significant difference in recovery time, there was a trend toward delayed recovery under the Box Breathing condition. These results suggest that controlled breathing techniques may not produce uniform physiological responses in all individuals and that their effects may vary depending on the type and intensity of exercise.

**Limitations and Future Studies**

One of the main limitations of this study is that it only evaluates the acute effects of breathing techniques. The effects of long-term applications (e.g., 4–8 weeks) on autonomic nervous system adaptations are unknown. Chronic studies should monitor changes in parameters such as HR recovery and HRV and include additional physiological markers such as baroreflex sensitivity (Dupuy et al., 2018). Additionally, the use of respiratory rate monitors to standardise participants' breathing patterns would enhance data reliability.

Future research should compare the effects of controlled breathing protocols across different exercise intensities and durations.

Different age groups and resting heart rate levels may provide different assessments and perspectives on the evaluation of heart rate recovery (HRR) and parasympathetic activity.

**Edited version**

**Updates and corrections have been made in the relevant sections, taking into account the warnings and suggestions.**

4 **Discussion**

The aim of this study was to compare the effects of three different breathing protocols (spontaneous breathing, box breathing, and 6 bpm) on heart rate, recovery time, and perceived exertion after high-intensity interval training (HIIT) on a spin bike. The findings revealed that the Box Breathing protocol resulted in significantly higher post-exercise heart rates and perceived exertion scores compared to the other protocols. These results indicate that the choice of breathing technique can significantly influence both physiological and perceptual responses during post-exercise recovery.

Contrary to the typical parasympathetic activation associated with slow breathing techniques [1,19], the Box Breathing protocol elicited a significantly higher post-exercise heart rate compared to both 6 bpm and spontaneous breathing. A potential explanation for this paradoxical response lies in the unique structure of box breathing, which incorporates breath-holds. During the high metabolic demand following HIIT, these apneic phases may have induced transient hypercapnia, thereby stimulating a sympathetic response [20] a mechanism supported by findings that breath-holding can temporarily reduce heart rate variability post-exercise [19]. This suggests that the autonomic effects of breathing techniques are context-dependent and can be significantly modulated by exercise intensity. Future research incorporating direct measurements of end-tidal CO₂ and heart rate variability is warranted to confirm this hypothesis.

The observed differences were substantiated by large effect sizes for both final heart rate (η² = 0.127; Box vs. 6 bpm Cohen's d = 0.907) and perceived exertion (η² = 0.374; Box vs. Spont. d = 1.525, Box vs. 6 bpm d = 1.694), underscoring the substantial physiological and perceptual impact of the box breathing technique. In contrast, no significant difference was found in recovery time to baseline heart rate between protocols, which contrasts with studies reporting facilitatory effects of similar techniques on recovery [21,23]. This discrepancy may be attributed to differences in exercise intensity, participant characteristics, or protocol duration, highlighting the need for future studies with standardized long-term applications on homogeneous groups to clarify these effects. The moderate effect size for recovery time between Box Breathing and 6 bpm (Cohen's d = 0.502), though statistically non-significant, warrants further investigation into the potential benefits of rhythmic breathing like 6 bpm.

The 6 bpm protocol, a form of resonance frequency breathing known to enhance cardiorespiratory synchronization [2,13], resulted in heart rate and perceived exertion levels comparable to spontaneous breathing. This aligns with previous reports that slow breathing may not invariably accelerate physiological recovery post-exercise [9], suggesting its primary benefit in this context may not be superior cardiovascular restoration compared to natural breathing.

A particularly striking finding was the substantially higher perceived exertion following Box Breathing, supported by a very large effect size (η² = 0.374). The breath-hold components of this technique, performed under the high physiological stress of post-HIIT recovery, likely contributed to increased psychological and physical strain [14,26]. This contrasts with studies conducted in low-stress or low-intensity conditions [2], underscoring that the perceptual impact of breathing techniques is highly sensitive to the concurrent physiological state.

Finally, there are only a limited number of studies in the literature that directly evaluate the acute effects of breathing techniques on post-exercise physiological recovery [11,15]. Additionally, Garcia et al. [27] highlighted that individuals with lower resting heart rate (RHR) exhibited faster HRR and stronger parasympathetic reactivation, while Danieli et al. [28] emphasized that resting HRV values in middle-aged individuals were significantly positively correlated with early HRR post-exercise. These findings draw attention to the effects of resting heart rate and age on autonomic regulation and suggest the need for age-specific analyses.

In this context, our study contributes significantly to the field by comparing the effects of controlled breathing techniques on post-exercise heart rate and perceived exertion in young, physically active individuals.

5. **Conclusions and Recommendations**

The results of this study strengthen the assessment of different breathing protocols as indicators that may create significant differences in physiological recovery and perceived exertion after high-intensity interval training (HIIT).

**Compared to Spontaneous Breathing:**

- The Box Breathing protocol resulted in higher heart rate and perceived exertion.
- The 6 bpm protocol led to lower physiological and perceptual loads.
- Although there was no significant difference in recovery time, there was a trend toward delayed recovery under the Box Breathing condition.

These results suggest that controlled breathing techniques may not produce uniform physiological responses in all individuals and that their effects may vary depending on the type and intensity of exercise.

**Limitations and Future Studies**

This study has several limitations that should be considered. Firstly, the findings are constrained by their acute nature, capturing only the immediate effects of a single application of the breathing protocols. The long-term adaptations and efficacy of sustained practice (e.g., over 8-12 weeks) remain unknown and represent a crucial area for future investigation.

Secondly, the generalizability of the results is limited by the specific sample characteristics. The participants were young, healthy, and physically active sports science students. Consequently, these findings may not be directly applicable to older adults, elite athletes, sedentary individuals, or populations with different fitness levels or health conditions. The effectiveness of these breathing strategies is likely influenced by factors such as age, baseline fitness, and training status, which warrants further exploration.

To address these limitations, future chronic studies should monitor changes in parameters such as HR recovery and heart rate variability (HRV), incorporating additional physiological markers like baroreflex sensitivity [3]. Furthermore, research should compare the effects of these protocols across different exercise intensities, durations, and population groups. The use of respiratory rate monitors to standardize breathing patterns would also enhance the reliability of future data.

**Practical Implications**

The findings of this study offer valuable practical applications for coaches, athletes, and fitness professionals. According to our results, the 6 breaths/min breathing protocol (5-second inhale, 5-second exhale) is a more effective and less stressful strategy for post-HIIT recovery compared to Box Breathing (4-4-4-4). This technique can be easily implemented in training settings: coaches can instruct athletes to begin the 6 breaths/min rhythm during rest intervals between sets or immediately upon finishing a session, using simple verbal cues ("Breathe in... two... three... four... five... Now breathe out... two... three... four... five...") or a visual metronome to maintain the pace. The protocol's simplicity allows for rapid adoption without the need for specialized equipment. In contrast, practitioners should be cautious when prescribing Box Breathing immediately after high-intensity exercise, as it may inadvertently increase physiological and perceptual stress, potentially delaying recovery. For optimal results, the regular application of the 6 breaths/min technique during inter-set rest periods or post-training is recommended.

d) **Expand the discussion on the practical applicability of respiratory protocols.**

**Previous version**

**Conclusions and Recommendations**

The results of this study strengthen the assessment of different breathing protocols as indicators that may create significant differences in physiological recovery and perceived exertion after high-intensity interval training (HIIT).

**Compared to Spontaneous Breathing:**

- The Box Breathing protocol resulted in higher heart rate and perceived exertion.
- The 6 Breaths/Minute protocol led to lower physiological and perceptual loads.
- Although there was no significant difference in recovery time, there was a trend toward delayed recovery under the Box Breathing condition. These results suggest that controlled breathing techniques may not produce uniform physiological responses in all individuals and that their effects may vary depending on the type and intensity of exercise.

**Limitations and Future Studies**

One of the main limitations of this study is that it only evaluates the acute effects of breathing techniques. The effects of long-term applications (e.g., 4–8 weeks) on autonomic nervous system adaptations are unknown. Chronic studies should monitor changes in parameters such as HR recovery and HRV and include additional physiological markers such as baroreflex sensitivity (Dupuy et al., 2018). Additionally, the use of respiratory rate monitors to standardise participants' breathing patterns would enhance data reliability.

Future research should compare the effects of controlled breathing protocols across different exercise intensities and durations.

Different age groups and resting heart rate levels may provide different assessments and perspectives on the evaluation of heart rate recovery (HRR) and parasympathetic activity.

**Edited version**

The "Practical Applicability" description identified and suggested has been added as a subheading to the Discussion section.

**Conclusions and Recommendations**

The results of this study strengthen the assessment of different breathing protocols as indicators that may create significant differences in physiological recovery and perceived exertion after high-intensity interval training (HIIT).

**Compared to Spontaneous Breathing:**

- The Box Breathing protocol resulted in higher heart rate and perceived exertion.
- The 6 bpm protocol led to lower physiological and perceptual loads.
- Although there was no significant difference in recovery time, there was a trend toward delayed recovery under the Box Breathing condition.

These results suggest that controlled breathing techniques may not produce uniform physiological responses in all individuals and that their effects may vary depending on the type and intensity of exercise.

**Limitations and Future Studies**

This study has several limitations that should be considered. Firstly, the findings are constrained by their acute nature, capturing only the immediate effects of a single application of the breathing protocols. The long-term adaptations and efficacy of sustained practice (e.g., over 8-12 weeks) remain unknown and represent a crucial area for future investigation.

Secondly, the generalizability of the results is limited by the specific sample characteristics. The participants were young, healthy, and physically active sports science students. Consequently, these findings may not be directly applicable to older adults, elite athletes, sedentary individuals, or populations with different fitness levels or health conditions. The effectiveness of these breathing strategies is likely influenced by factors such as age, baseline fitness, and training status, which warrants further exploration.

To address these limitations, future chronic studies should monitor changes in parameters such as HR recovery and heart rate variability (HRV), incorporating additional physiological markers like baroreflex sensitivity [3]. Furthermore, research should compare the effects of these protocols across different exercise intensities, durations, and population groups. The use of respiratory rate monitors to standardize breathing patterns would also enhance the reliability of future data.

**Practical Implications**

The findings of this study offer valuable practical applications for coaches, athletes, and fitness professionals. According to our results, the 6 breaths/min breathing protocol (5-second inhale, 5-second exhale) is a more effective and less stressful strategy for post-HIIT recovery compared to Box Breathing (4-4-4-4). This technique can be easily implemented in training settings: coaches can instruct athletes to begin the 6 breaths/min rhythm during rest intervals between sets or immediately upon finishing a session, using simple verbal cues ("Breathe in... two... three... four... five... Now breathe out... two... three... four... five...") or a visual metronome to maintain the pace. The protocol's simplicity allows for rapid adoption without the need for specialized equipment. In contrast, practitioners should be cautious when prescribing Box Breathing immediately after high-intensity exercise, as it may inadvertently increase physiological and perceptual stress, potentially delaying recovery. For optimal results, the regular application of the 6 breaths/min technique during inter-set rest periods or post-training is recommended.

e) **Reinforce the limitations related to the sample and the acute nature of the observed effects.**

**Previous version**

**Limitations and Future Studies**

One of the main limitations of this study is that it only evaluates the acute effects of breathing techniques. The effects of long-term applications (e.g., 4–8 weeks) on autonomic nervous system adaptations are unknown. Chronic studies should monitor changes in parameters such as HR recovery and HRV and include additional physiological markers such as baroreflex sensitivity (Dupuy et al., 2018). Additionally, the use of respiratory rate monitors to standardise participants' breathing patterns would enhance data reliability.

Future research should compare the effects of controlled breathing protocols across different exercise intensities and durations.

Different age groups and resting heart rate levels may provide different assessments and perspectives on the evaluation of heart rate recovery (HRR) and parasympathetic activity.

**Edited version**

**Limitations and Future Studies**

This study has several limitations that should be considered. Firstly, the findings are constrained by their acute nature, capturing only the immediate effects of a single application of the breathing protocols. The long-term adaptations and efficacy of sustained practice (e.g., over 8-12 weeks) remain unknown and represent a crucial area for future investigation.

Secondly, the generalizability of the results is limited by the specific sample characteristics. The participants were young, healthy, and physically active sports science students. Consequently, these findings may not be directly applicable to older adults, elite athletes, sedentary individuals, or populations with different fitness levels or health conditions. The effectiveness of these breathing strategies is likely influenced by factors such as age, baseline fitness, and training status, which warrants further exploration.

To address these limitations, future chronic studies should monitor changes in parameters such as HR recovery and heart rate variability (HRV), incorporating additional physiological markers like baroreflex sensitivity [3]. Furthermore, research should compare the effects of these protocols across different exercise intensities, durations, and population groups. The use of respiratory rate monitors to standardize breathing patterns would also enhance the reliability of future data.
